# Sustained Monomorphic Ventricular Tachycardia Accurately Detected by Wearable Technology

**DOI:** 10.1016/j.jaccas.2025.103273

**Published:** 2025-03-12

**Authors:** Nicole Guynn, Kierra Regis, Alex Eckert, Melroy D’souza, Sarah Turbow, Stacy Westerman, Laurence Sperling, Michael Lloyd

**Affiliations:** Division of Cardiology, Department of Medicine, Emory University School of Medicine, Atlanta, Georgia, USA

**Keywords:** palpitations, smart watch, ventricular arrhythmia, wearable technology, wide complex tachycardia

## Abstract

Wearable devices are increasingly used to detect arrhythmias, including life-threatening ventricular tachycardia (VT). This case highlights their role in clinical settings. A patient with prior surgical aortic valve replacement because of a bicuspid valve experienced symptomatic palpitations, and his smartwatch recorded a wide complex tachycardia at 165 beats/min. His presentation led to intensive care admission and later to a diagnosis of VT successfully treated with an implantable defibrillator and ultimately requiring radiofrequency ablation. Smartwatches provide a noninvasive tool for detecting arrhythmias like VT, using photoplethysmography and single-lead ECG. Limitations include validation gaps, false positives, and user activation. Integration into clinical practice requires updated guidelines, structured reimbursement, and legal clarity. This case demonstrates the critical role of smartwatch data in VT detection and management.

## Graphical Abstract

**Figure:**
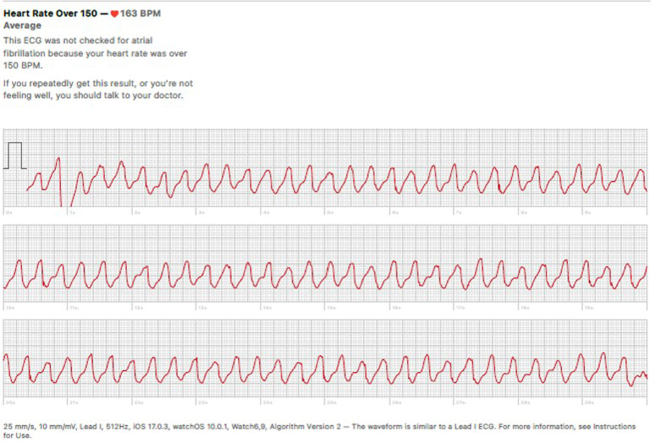

A 64-year-old man with a history of surgical bioprosthetic aortic valve (AV) replacement at age 55 presented to his primary care clinician describing intermittent palpitations and near-syncope over the previous month. The symptoms corresponded to a wide complex tachycardia with a rate of 165 beats/min on his smartwatch (Figure 1). On admission, the patient was in sinus rhythm with a heart rate of 77 beats/min. His blood pressure was 147/90 mm Hg, his temperature was 98.6 °F, and his oxygen saturation was 99% on room air. The result of his cardiac examination was notable for an early peaking systolic murmur without a diastolic murmur. The patient was admitted to the telemetry unit but later transferred to the intensive cardiac unit because of multiple episodes of monomorphic ventricular tachycardia (VT) captured by telemetry and ECG (Figure 2). These episodes resulted in symptoms similar to those he felt at home.

**Take-Home Message**

• Smartwatch data can be crucial in the early detection and management of life-threatening arrhythmias like ventricular tachycardia, enabling timely clinical intervention that might otherwise be delayed.

**Figure 1:**
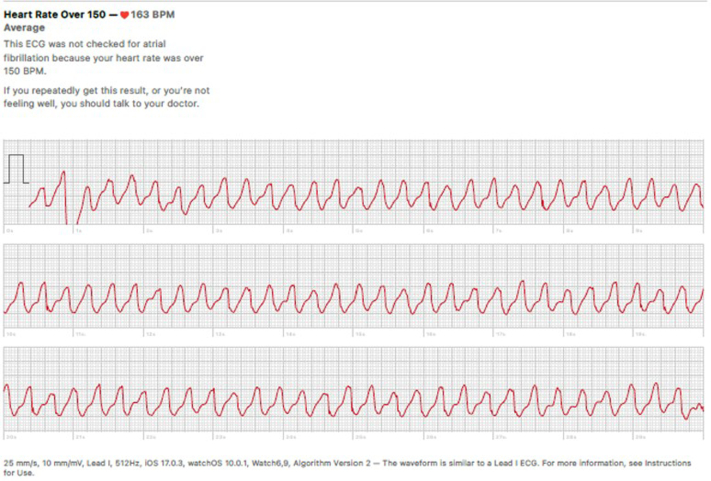
Monomorphic Ventricular Tachycardia Captured by Patient With Personal Wearable Device

**Figure 2:**
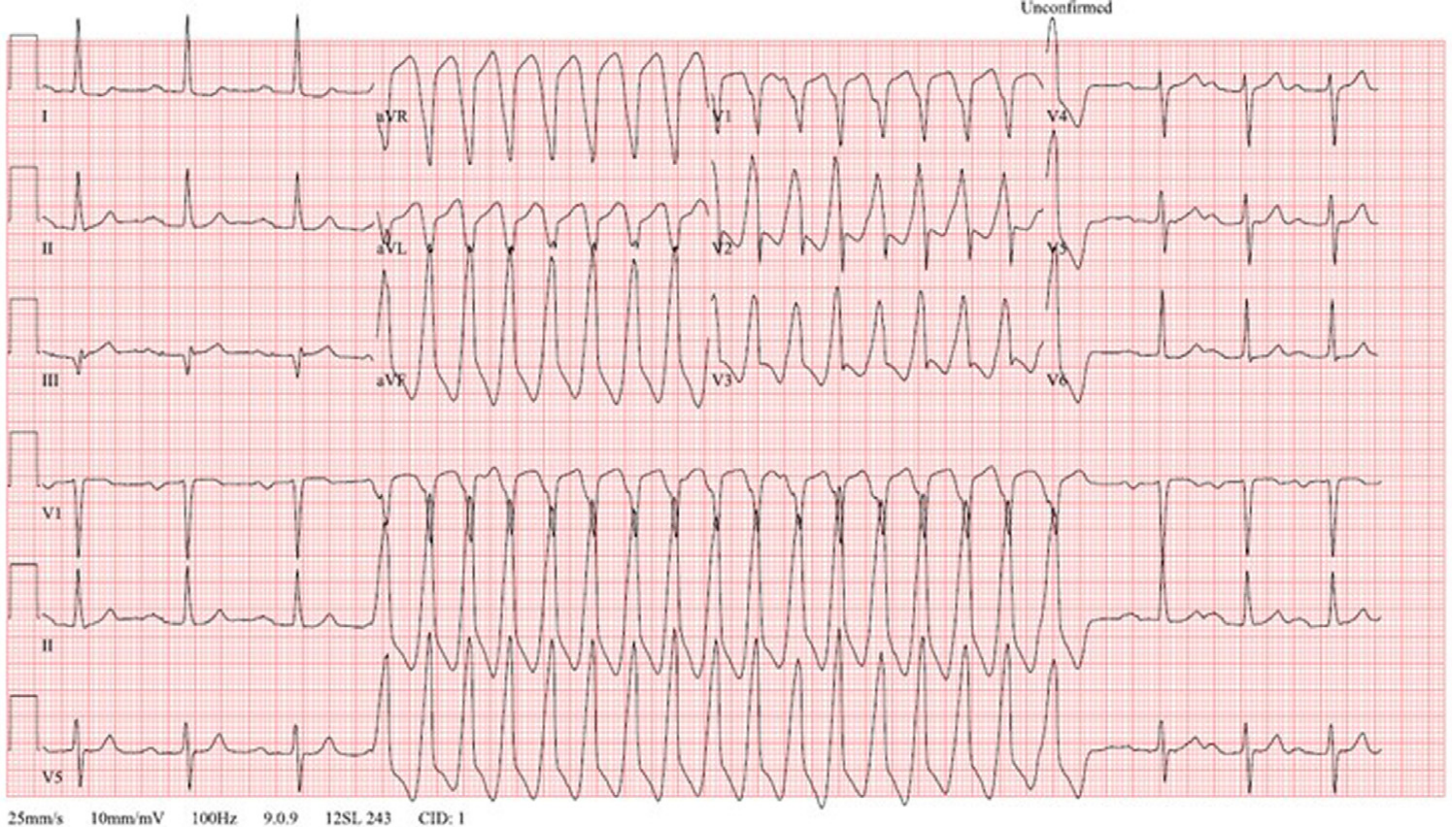
Representative Ventricular Tachycardia During Initial Evaluation as Seen on 12-Lead Electrocardiogram The vector suggests an exit site near the left ventricular outflow tract region.

## Past Medical History

His medical history included hyperlipidemia, obstructive sleep apnea, gastroesophageal reflux disease, and coronary artery disease (CAD) valve replacement. His mother had died at age 92 of cancer-related complications but also had heart disease and hypertension. His father had hypertension and CAD, had a pacemaker, and had no known genetic conditions. One of his children had died in sleep of a cerebral aneurysm. The others were in good health. The patient exercised regularly 3 or 4 times a week for at least 30 to 45 minutes at a time and did not describe smoking, heavy alcohol use, or illicit drug use.

## Differential Diagnosis

The differential diagnosis for wide complex tachycardia is short: monomorphic VT pre-excited tachycardia or aberrated supraventricular tachycardia. The aberrated supraventricular tachycardias could include AV nodal reentrant tachycardia, AV reciprocating tachycardia, or atrial tachycardia. Pre-excited tachycardia was less likely, given the normal resting ECG. The tracings captured on single lead followed by standard ECG demonstrated wide QRS with atrioventricular dissociation consistent with monomorphic VT by the Vereckei criteria. The differential of this sustained monomorphic VT included outflow tract tachycardia based on the morphology captured on 12 lead, ischemic VT due to age and risk factors, and other substrate-based tachycardias including those from myocarditis, cardiac sarcoidosis, or scar related to prior valve surgery.^1^, ^2^, ^3^

## Investigations

The patient’s laboratory results were notable for potassium 3.7 mEq/L, magnesium 1.8 mg/dL, brain natriuretic peptide 117 pg/mL, and elevated high-sensitivity troponin 13 ng/mL. In the intensive care unit, he was screened for coronavirus disease, flu, and respiratory syncytial virus. Echocardiography revealed left ventricular (LV) ejection fraction (EF) of 50% to 55% with mildly increased wall thickness and a moderately dilatated right ventricle (RV) with normal systolic function. The bioprosthetic AV was well seated, with a mean gradient and peak velocity of 20 mm Hg and 2.9 m/s, respectively. A coronary angiogram showed nonobstructive CAD. Cardiac magnetic resonance (CMR) was then performed for suspicion of myocarditis. CMR showed delayed gadolinium enhancement with possible edema at the basal septal LV, which suggested scar or late myocarditis in a region compatible with the origin of VT. CMR also showed mildly depressed LV and RV EF. Additional viral serology samples were sent for evaluation as potential causes of myocarditis, and the result of Coxsackie B antibody titer was mildly positive.

## Medical Intervention

The patient was given metoprolol 25 mg twice a day, which was increased to 50 mg twice a day because of frequent VT episodes 20 to 25 beats long with sustained episodes captured on telemetry. The result of a diagnostic EP study was notable for easily inducible VT with morphology that suggested LV outflow tract exit sites. Also, the clinical arrhythmia on the smartwatch was symptomatic and sustained. On EP study, the VT was sustained and pace terminable. In accordance with the 2008 ACC/AHA/HRS guidelines for device-based therapy, which stated that ICD therapy is indicated in patients with structural heart disease and spontaneous sustained VT, whether hemodynamically stable or unstable, there was a Class I indication for secondary prevention ICD in this patient with a history of structural heart disease requiring surgical valve replacement and sustained symptomatic VT.^4^ Whereas it was arguable that an ablation rendering him noninducible might have eliminated his need for an ICD, he opted for ICD implantation during the index hospitalization and deferred a possible ablation at that time. A single-chamber transvenous secondary prevention implantable defibrillator was placed with the intent to perform future ablation. Given his reduced EF, the patient was given spironolactone and dapagliflozin in addition to his home use of valsartan. He was discharged when his VT abated with medical treatment alone.

**Figure 3:**
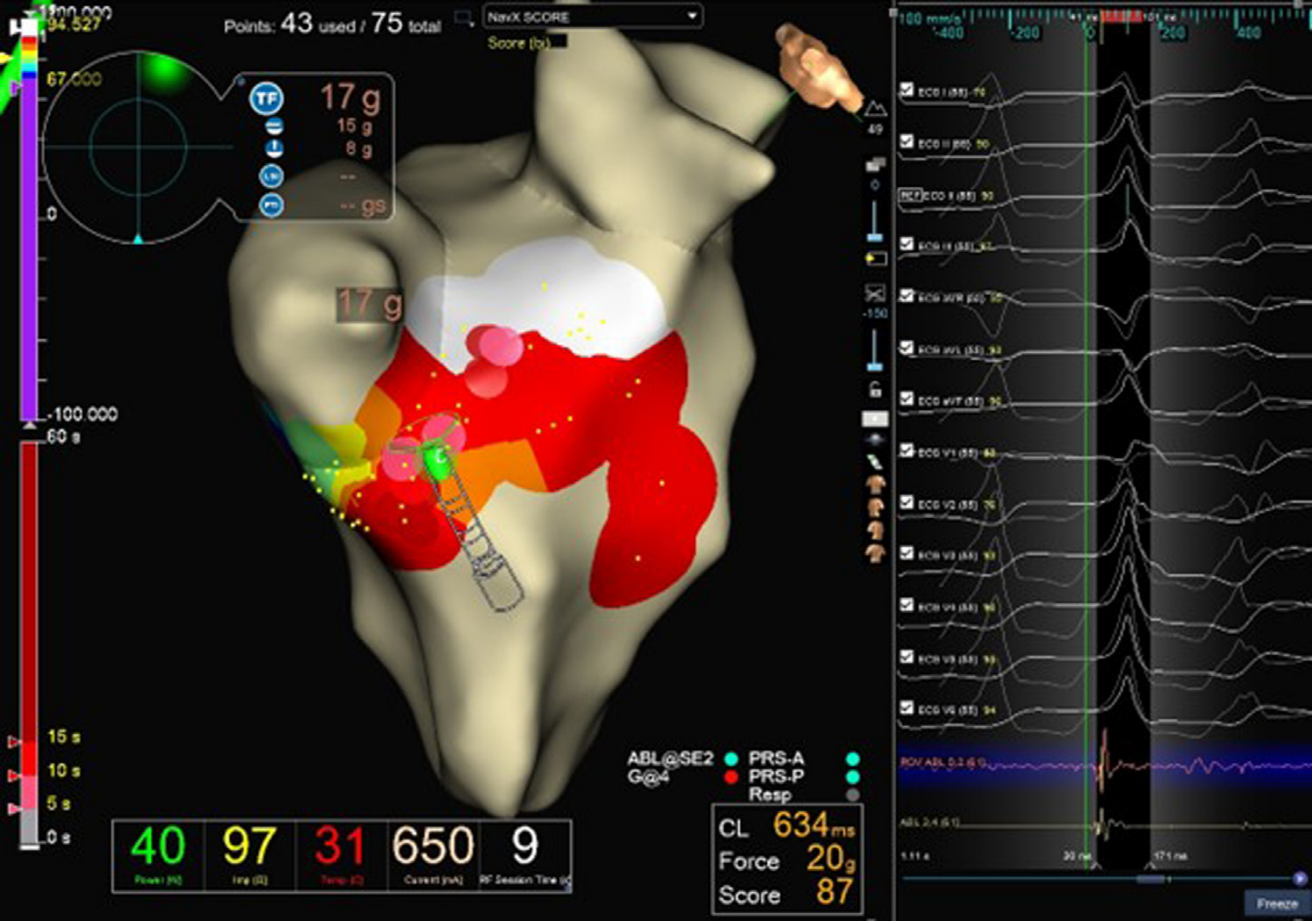
Site of Successful Radiofrequency Ablation of Ventricular Tachycardia Below Aortic Bioprosthesis Red dots, ablation lesions.

**Figure 4:**
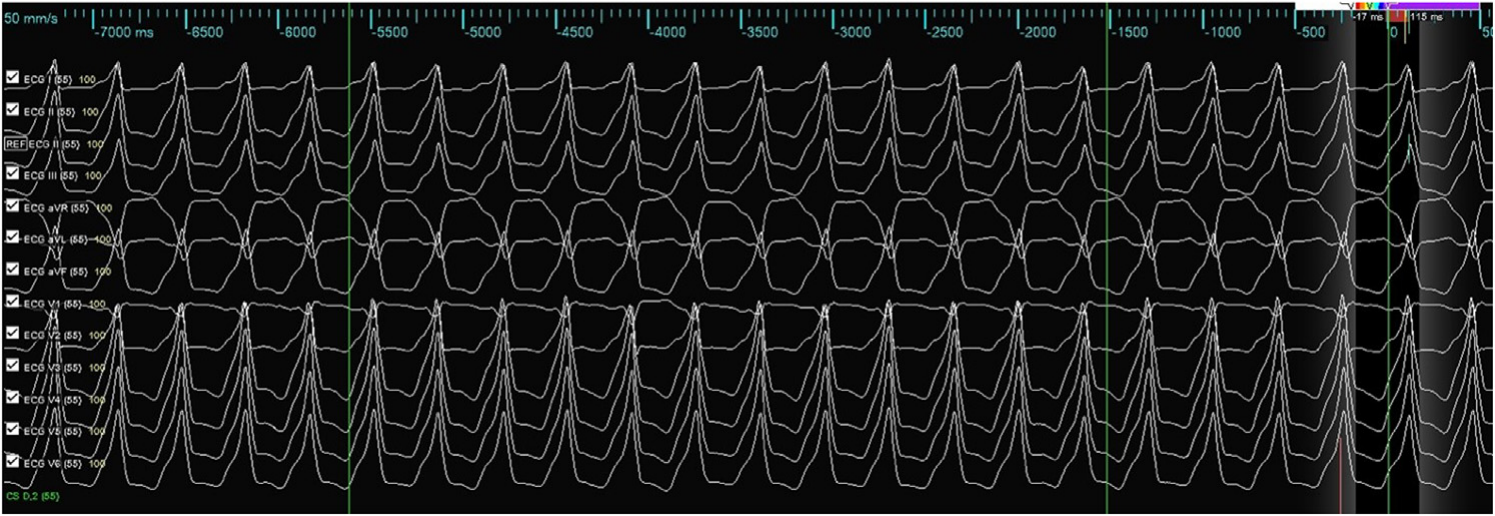
12-Lead Electrocardiogram of Inducible Ventricular Tachycardia Before Ablation

Three months following discharge he was shocked by his implantable defibrillator and interrogation showed VT. Review of his device revealed that he had many pace-terminated VT episodes prior to episodes several of which degenerated into ventricular fibrillation leading to successful implantable defibrillator shocks. The patient received an ICD shock for a much faster VT months after implant indicating that the decision to place an ICD was reasonable. The patient underwent successful ablation following induction of an outflow tract tachycardia at the mitral aspect of the AV prosthesis at a region near the aorto-mitral continuity (Figures 3 and 4). The ablation was performed via a transseptal approach, given the AV, using multi-electrode mapping catheters and force-sensing irrigated ablation catheters with powers of up to 40 watts. The patient had no postoperative complications. The working diagnosis was scar-based VT below the AV bioprosthesis.

## Discussion

The surging ownership of smartwatches highlights an opportunity and a challenge in clinical practice. These devices could promptly diagnose and manage life-threatening arrhythmias in patients experiencing transient symptoms, but the sheer volume of data a clinician might be presented with could overwhelm the healthcare system.^5^ With features like history tracking, health notifications, and the ability to share data electronically with providers, wearable devices can provide information to clinicians that can aid in clinical decision making. The smartwatch offers a noninvasive approach to arrhythmia detection in comparison with some of the other tools available. Current tools used for ambulatory arrhythmia monitoring primarily use 3 technologies: photoplethysmography (PPG), ECG, and mechanocardiography.^6^ Of the 3 technologies, PPG is used the most in devices like the Apple Watch, the Samsung Galaxy Watch, and the Fitbit. In particular, the Apple Watch irregular pulse detection algorithm was found to have a positive predictive value of 0.84 for identification of AF.^7^ In a recent study evaluating arrhythmias other than AF detected with the Apple Watch irregular pulse algorithm, any arrhythmia (excluding supraventricular tachycardia <30 beats and pauses <3 seconds) was detected in 119 of 297 participants (40.1%). These arrhythmias included atrial and ventricular ectopy and heart block.^8^ There are concerns regarding their validation beyond atrial fibrillation (AF) along with false positive readings and the associated potential to exacerbate patient anxiety without substantial outcome improvements.^9^ Any wearable device with single-lead ECG technology would be able to capture evidence of VT, including the Apple Watch, Samsung Watch, and Fitbit. Although none of these algorithms are validated to detect VT, single-lead ECGs collected by wearable technologies can lead clinicians to earlier diagnosis of arrhythmias through symptom rhythm correlation and review of QRS morphology.

In our case, the patient activated the ECG function on his smartwatch while experiencing palpitations and near syncope. The watch captured a 10-second single-lead ECG and reported a heart rate >150 beats/min with a warning to speak to a doctor if the rate was persistent or if the wearer was not feeling well. Presenting these recordings to a cardiologist allowed for earlier intervention. To date, there have only been 3 other reported cases of VT diagnosed by a smartwatch. Each one outlines a similar scenario where an individual has symptomatic tachycardia in the field captured only by a smartwatch because of its transient nature. All these patients underwent an EP study leading to implantable defibrillator placement, ablation, or both.^10^

Despite improvements in understanding how to use smartwatch data in clinical settings, significant gaps remain. Compared with arrhythmia-detecting devices like implantable loop recorders or Holter-type patch ECGs, smartwatches are limited by a single-lead ECG and will require follow-up imaging to confirm QRS morphology. They do not have a continuous ECG function and must be triggered by the user to record a tracing. There is no standardized method to evaluate smartwatch data in the healthcare setting, and no structured reimbursement system. Clinicians will need to consider updating guidelines and supporting physicians in navigating legal considerations regarding the incorporation or dismissal of wearable device data. Nonetheless, the data gleaned from smartwatches holds promise in capturing arrhythmias that may otherwise go undetected and in guiding clinical decisions.

## Follow-Up

After the patient underwent ablation, serial device interrogations showed no further VT episodes and no deterioration of his LVEF.

## Conclusions

This case highlights the potential value of smartwatch data during symptom-triggered evaluation of symptomatic palpitations. There is a need for a more cost-effective approach to evaluation and management of palpitations, and this case demonstrates that VT would have gone undiagnosed if this patient’s clinician had not considered the smartwatch data. As wearable technology continues to evolve, clinical practice guidelines may need to evolve to incorporate smartwatch data into the management of palpitations.

## Funding Support and Author Disclosures

Dr Lloyd has received consultant fees from BoSci Medtronic, Abbott, and honoraria relationships. All other authors have reported that they have no relationships relevant to the contents of this paper to disclose.
